# Revisiting ventilator-induced lung injury: from mechanical power to immunometabolic interaction

**DOI:** 10.3389/fimmu.2026.1754983

**Published:** 2026-01-22

**Authors:** Yuelian Luo, Zixuan Wu, Jinglei Wu, Zijian You, Yue Chen, Xinquan Wei, Yujie Liang, Jiancong Yang, Mingbo Luo, Jie Chen, Huijun Dai

**Affiliations:** Department of Anesthesiology, Liuzhou People’s Hospital Affiliated to Guangxi Medical University, Liuzhou, Guangxi, China

**Keywords:** lung injury, mechano-immuno-metabolic, molecular mechanisms, targeted strategies, ventilator-induced lung injury

## Abstract

Ventilator-induced lung injury (VILI) occurs through the interaction of mechanical stress, immune system changes, and metabolic shifts. Models that focus mainly on pressure and volume do not fully represent this complex process. Recent work shows that mechanical power provides a way to measure energy load and its relationship to biological injury. When mechanical energy becomes excessive, it activates cellular sensing mechanisms, leads to mitochondrial dysfunction, and activates immune responses, which increases inflammation and damages tissue. This review synthesizes recent findings regarding the interplay among mechanical, immune, and metabolic factors, examining how energy transfer, cellular signaling, and shifts in metabolic function contribute to the development of ventilator-induced lung injury. New approaches using immune-modifying treatments, compounds that reduce oxidative damage (e.g., hesperidin and exogenous surfactant), and therapies using particles from stem cells offer possible directions for improved ventilation methods and protection of lung tissue.

## 1 Introduction

Ventilator-induced lung injury (VILI) is a significant clinical concern arising from mechanical ventilation, leading to inflammatory damage primarily due to mechanical factors affecting the lungs. This lung injury is characterized by a bioenergetic-immune disorder resulting from the inappropriate transfer of mechanical energy during ventilation, which can compromise gas exchange and promote pathological responses (1–3). The conventional “four-fold injury” model elucidates the mechanisms behind VILI, categorizing the underlying pathophysiological processes into four types: volumetric injury (volutrauma), barotrauma, atelectrauma, and biotrauma. Each of these mechanisms contributes to the inflammatory cascade seen in VILI, where excessive mechanical stress leads to cellular injury and promotes the infiltration of inflammatory cells, cytokine release, and oxidative stress (2, 4–6). Recent studies indicate that energy-based models facilitate quantifying the extent of VILI through mechanical power metrics, which encapsulate key ventilatory parameters such as tidal volume, driving pressure, respiratory rate, and positive end-expiratory pressure (PEEP) into a singular value expressed in joules per minute (J/min) (3, 7, 8). Mechanical power exceeding a threshold of 12–15 J/min poses substantial risks, including cell damage, extracellular matrix degradation, and enhanced systemic inflammatory response (9). Critically, even with lung-protective ventilation strategies, patients may still experience exacerbation of pre-existing lung injuries due to cumulative mechanical power, contributing to elevated mortality rates in intensive care settings (10).

Clinically, VILI manifests through various pathophysiological indicators, including progressive disruption of the alveolar-capillary barrier, reduced lung compliance, and worsening oxygenation, as evidenced by imaging findings such as ground-glass opacities in radiographs (11–13). Laboratory assessments frequently reveal elevated inflammatory markers in bronchoalveolar lavage fluid, highlighting the inflammatory nature of the condition (14, 15).

The incidence of VILI remains high in Acute Respiratory Distress Syndrome (ARDS) patients, with a mortality rate exceeding 40%. It also constitutes a significant clinical burden in perioperative ventilation patients, especially in elderly patients with underlying lung diseases (1, 16, 17). Epidemiological studies show that the in-hospital mortality rate of ARDS patients is as high as 41.1%, with VILI being a significant driver of death by inducing systemic inflammatory response and multiple organ failure (1, 18). In specific ICU populations, VILI resulted in an average extension of 7.3 days of mechanical ventilation and a 2.4-fold increase in the risk of secondary infection, which has an additive damage effect on sepsis patients (19); studies on surgical anesthesia patients showed that the incidence of VILI increased sharply to 62% when transpulmonary pressure was >20 cmH_2_O, and was significantly associated with postoperative cognitive impairment (20, 21). Due to decreased alveolar epithelial repair capacity and impaired mitochondrial function, the pathological severity of VILI is 37% higher in older patients compared to younger patients under the same mechanical parameters (16, 19). Therefore, understanding the energy and inflammatory mechanisms of lung injury during mechanical ventilation in VILI is of great significance for optimizing lung-protective ventilation strategies and reducing patient mortality and the risk of complications.

In recent years, research on VILI has been undergoing a shift from traditional single-parameter optimization to a new paradigm that integrates energy and immune-metabolic factors. This review, based on these research advances, aims to provide a cognitive framework and clinical reference for the multidimensional mechanism research and personalized treatment strategies of VILI by systematically integrating the latest evidence in the field.

## 2 Molecular pathophysiology

### 2.1 Mechanical power and energy load model

Central to understanding VILI is the concept of mechanical power (MP), which represents the total energy transferred from the ventilatory apparatus to the respiratory system per minute. This energy is composed of three primary components: elastic work due to lung compliance, work against airway resistance, and work associated with respiratory rate (1, 22, 23). The mechanical power equation, expressed as MP (J/min)=0.098×VT×RR×(Ppeak - ½ ΔP)(where VT is tidal volume, RR is respiratory rate, Ppeak is peak pressure, and ΔP is driving pressure), highlights that it quantifies the energy transferred during a single breath, incorporating both static and dynamic pressures, and multiplying by the respiratory rate (23, 24). This energy transfer can be categorized into conservative energy (elastic potential energy) and dissipative energy (energy lost due to airflow). The majority of evidence suggests that the elastic component, often linked to lung inflammation, significantly contributes to exacerbating lung injuries (21, 24).

It is essential to note that adjusting individual ventilatory parameters alone often does not suffice to reduce the risk of VILI; the cumulative energy represented by MP is a more reliable predictor of biological injury than isolated metrics such as tidal volume or driving pressure (25). Challenges persist in the clinical adoption of MP metrics owing to difficulties in computational standardization and the determination of individualized therapeutic thresholds (26, 27).

In summary, MP is a crucial factor in the context of VILI. A comprehensive evaluation must consider the synergistic effects of multiple ventilatory parameters, emphasizing the need for improved analytical methods to enhance clinical outcomes in mechanically ventilated patients (28, 29).

### 2.2 Mechanical signal transduction and cellular response

Mechanical signal transduction is a critical physiological process that involves the sensing of mechanical stimuli and the subsequent cellular responses. One of the key mechanisms through which this process occurs is via mechanosensitive ion channels, particularly the Piezo1 and Piezo2 channels. These channels are integral to the transduction of mechanical stretching into biochemical signals that alter cellular functions. Upon mechanical stretching, Piezo channels facilitate various cellular responses, including Ca²^+^ influx, which is pivotal for activating downstream signaling pathways such as NF-κB and MAPK (p38/JNK) (30, 31).

**Figure 1:**
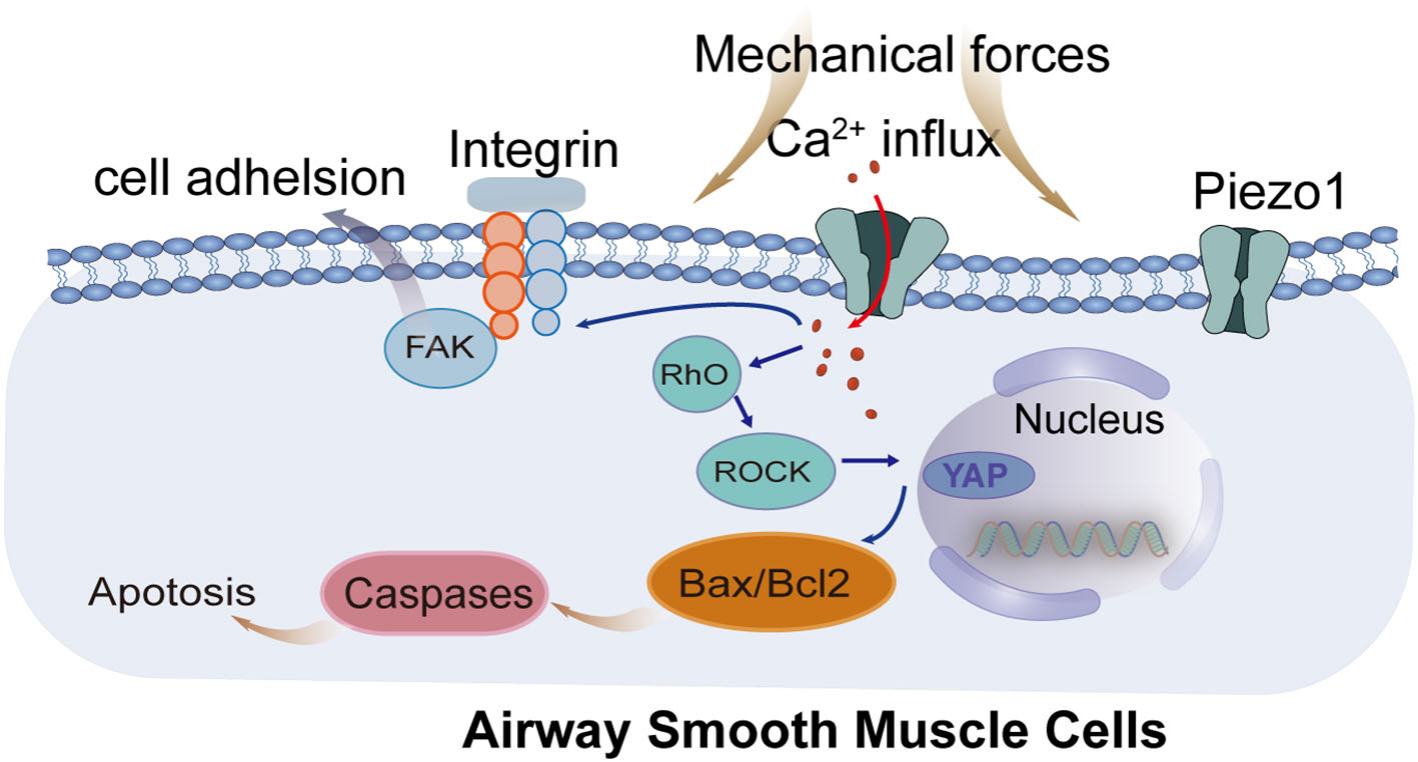
Signal transduction processes within airway smooth muscle cells under mechanical pressure. Mechanical forces are sensed by integrins and ion channels, triggering downstream signaling cascades involving cytoskeletal remodeling and mitochondrial dysfunction, ultimately leading to apoptosis. FAK, Focal Adhesion Kinase; Rho, Ras homolog gene family; ROCK, Rho-associated coiled-coil containing protein kinase; YAP, Yes-associated protein; Bax, Bcl-2-associated X protein; Bcl2, B-cell lymphoma 2; ROS, Reactive Oxygen Species; Piezo1, Piezo-type mechanosensitive ion channel component 1; TRPV4, Transient Receptor Potential Vanilloid 4.

The activation of the integrin-FAK-Src complex, in conjunction with these mechanosensitive channels, underscores a complex mechanism of cellular activation where Tyr925 phosphorylation plays a significant role (32). This phosphorylation is critical for mediating cellular adhesion and signaling, influencing a range of cellular responses including apoptosis in airway smooth muscle cells (**Figure 1**). Specifically, the Piezo1-RhoA/ROCK1 axis has been implicated in increasing the rates of apoptosis in these cells through mechanisms that disrupt junction complexes within vascular endothelial tissue, thus enhancing inflammatory responses (31, 33).

Activated alveolar epithelial cells and alveolar macrophages release IL-1β via the NLRP3 inflammasome, promote IL-6 secretion via the TLR4-p38 axis, and increase TNF-α (18, 34, 35). Simultaneously, the mechanical stress experienced by airway cells also prompts the increased expression of surface adhesion molecules including E-selectin, P-selectin, and ICAM-1, facilitating neutrophil migration by significant factors. The resultant inflammatory microenvironment can create a feedback loop conducive to further lung tissue compromise—evidenced by conditions such as atelectrauma, where the required opening pressures increase due to a reduction in surfactant. This synergistic effect leads to accelerated regional damage and complications such as volutrauma, where inflammatory mediators can be released in increased quantities (36, 37).

### 2.3 Oxidative stress and mitochondrial dysfunction

**Figure 2:**
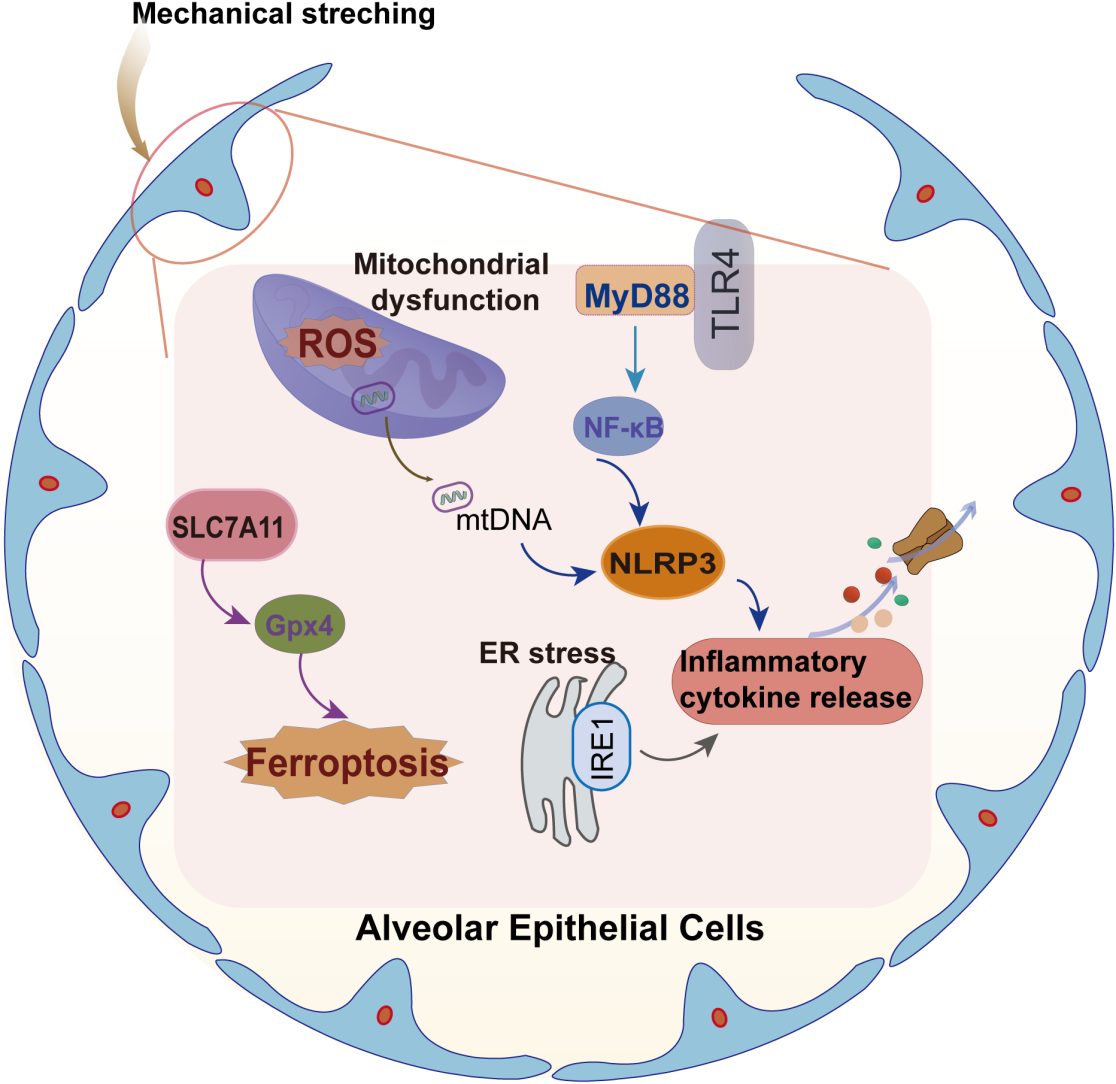
Schematic illustration of signal transduction and cellular response Induced by mechanical stretch in alveolar epithelial cells. Mechanical stretching triggers mitochondrial dysfunction and reactive oxygen species (ROS) generation, promoting mitochondrial DNA (mtDNA) release and activation of the TLR4–MyD88–NF-κB signaling axis. These events drive inflammatory cytokine release and NLRP3 inflammasome activation. In parallel, mechanical stretch induces endoplasmic reticulum (ER) stress and disrupts antioxidant defense by affecting the SLC7A11–GPX4 axis, leading to lipid peroxidation and ferroptosis in alveolar epithelial cells. TLR4, Toll-like receptor 4; MyD88, Myeloid differentiation primary response 88; NF-κB, Nuclear factor kappa B; ROS, Reactive oxygen species; mtDNA, Mitochondrial DNA; NLRP3, NLR family pyrin domain containing 3; ER, Endoplasmic reticulum; SLC7A11, Solute carrier family 7 member 11 (xCT); GPX4, Glutathione peroxidase 4.

Mechanical stretching and inflammatory signals (such as TNF-α/IL-1β) trigger a burst of reactive oxygen species (ROS) by activating the neutrophil NADPH oxidase complex and macrophage mitochondrial electron transport chain abnormalities (ETC complex III leakage increased by 58%) (1, 19, 38), High concentrations of ROS (·O_2_^-^/H_2_O_2_) lead to increase in mitochondrial DNA 8-hydroxydeoxyguanosine (8-OHdG) modification and decrease in mitochondrial membrane potential (ΔΨm) via the Fenton reaction. They also activate the TLR4-MyD88-NF-κB pathway (pro-inflammatory factor expression upregulation by 3.8-fold) and the NLRP3 inflammasome (caspase-1 activation level increased by 2.9-fold) via the mtDNA-CpG motif as a DAMP molecule (39) (**Figure 2**). This cascade results in several downstream physiological effects: 1) persistent opening of the mitochondrial permeability transition pore (mPTP) causes a reduction in ATP synthesis, tied to a concomitant drop in pyruvate dehydrogenase activity; 2) enhanced glycolytic metabolism driven by HIF-1α, as evidenced by an increase in lactate dehydrogenase (LDHA) expression; and 3) altered T cell differentiation, ^+^p;#x207A; T cells skew towards pro-inflammatory Th17 cell profiles, increasing their proportion while regulatory T cells (Tregs) diminish significantly. This “metabolic storm” engenders a feedback mechanism contributing to exacerbated immune dysregulation and further injury, thus establishing a vicious cycle of inflammation and oxidative damage (40–42). Experiments have shown that the use of MnTBAP (SOD mimic) can reduce mtDNA in bronchoalveolar lavage fluid in the VILI model by 47% and improve oxygenation index by 28%, highlighting the key therapeutic target value of this pathway (43).

### 2.4 Barrier damage and cell death

Mechanical stretching increases alveolar-capillary barrier permeability and induces protein-rich alveolar edema by promoting the degradation of the epithelial tight junction protein ZO-1 and the RAB7-dependent lysosomal degradation of endothelial VE-cadherin (44, 45). The multimodal coexistence of programmed cell death has been established: 1) Apoptosis is marked by the activation of Caspase-3/7 and an upregulation of the BAX/BCL-2 ratio, which correlates with the activation of the p38 MAPK-XOR axis; 2) Pyroptosis is mediated by the macrophage NLRP3-caspase-1-GSDMD axis, evidenced by a 4.2-fold increase in the GSDMD-NT fragment, which facilitates the maturation and release of IL-1β; 3) Ferroptosis is characterized by a 72% reduction in GPX4 protein expression, an accumulation of the lipid peroxidation product MDA, and a 58% decrease in mitochondrial cristae density, directly linked to the inhibition of the SLC7A11/GPX4 axis (39, 41, 46, 47). Zhang et al. demonstrated that macrophage-specific knockout of GSDMD reduces IL-18 levels in bronchoalveolar lavage fluid and improves lung tissue injury scores, underscoring the pivotal role of pyroptosis in the pathogenesis of VILI (40). Importantly, these three forms of cell death exhibit cross-regulation: Caspase-3 cleaves GPX4, thereby exacerbating ferroptosis, while mitochondrial ROS activates the NLRP3 inflammasome, promoting pyroptosis and forming a “death signaling network” that accelerates barrier collapse (48).

### 2.5 Immune response and inflammatory signaling

The immune response in VILI, often characterized as biotrauma, extends beyond a simple cytokine storm to involve complex, spatially distinct signaling networks triggered by mechanotransduction. Recent studies reveal that inflammatory mediators exhibit phase-dependent roles; for instance, TGF-β1, typically associated with fibrosis, has been shown to exert unexpected anti-inflammatory protection during the acute phase (49), whereas the IL6-Edn1-FoxO1 axis specifically links mechanical stress to structural lung growth arrest (50). This immune activation critically destabilizes the alveolar-capillary barrier, yet emerging interventions targeting endothelial integrity—such as the Tie2 agonist Vasculotide or modulation of sphingosine-1-phosphate (S1P) metabolism—have demonstrated efficacy in reducing permeability and pulmonary edema even under dual-hit conditions (51, 52). Furthermore, the immune landscape of VILI is topographically heterogeneous: advanced spatial transcriptomics indicate that inflammatory signatures are concentrated in atelectatic regions, distinguishing them from the cytoskeletal remodeling observed in overdistended zones (53). This underscores the necessity of viewing VILI immunity not as a uniform systemic reaction, but as a dynamic, regionally compartmentalized process driven by local mechanical forces.

## 3 Emerging therapeutic strategies

### 3.1 Mechanical energy management and individualized ventilation

The evolving paradigm of mechanical energy management in ARDS/ALI emphasizes lung-protective ventilation strategies anchored in three core principles: low tidal volume (<6 mL/kg predicted body weight) to mitigate volutrauma, driving pressure limitation (<15 cmH2O) to prevent regional overdistension, and mechanical power constraint (<12 J/min) as a composite measure integrating pressure-volume dynamics and respiration rate (1, 54, 55). Notably, von Düring’s 2025 meta-analysis demonstrated that maintaining mechanical power below 17 J/min reduces 28-day mortality by 38% (OR 0.62, 95%CI 0.51-0.75) through prevention of biotrauma-induced inflammatory cascades (8). Additionally, the concept of “mechanical intensity” introduces a multidimensional approach to ventilation management. This integrates global energy metrics with regional strain assessments, allowing for more personalized ventilation strategies (22, 47). Cutting-edge approaches integrate artificial intelligence with real-time physiological monitoring: Marini’s 2020 ventilation model predicts optimal inspiratory flow waveforms using deep reinforcement learning, while the IntelliVent-ASV® closed-loop system dynamically adjusts minute ventilation based on end-tidal CO2 kinetics, achieving 89% compliance with lung-protective targets in multicenter trials (47, 55). Emerging “energy-adaptive ventilation” platforms represent a significant advancement in mechanical ventilation technologies. By utilizing finite element analysis (FEA) to simulate parenchymal stress distributions, these systems enhance the understanding and management of mechanical ventilation, particularly in patients at risk for VILI. The concept involves integrating strain-limiting algorithms with esophageal pressure monitoring, leading to notable reductions in biomarkers associated with ventilator-induced lung injury according to pilot studies, although specific percentages require further validation from robust clinical trials (56).

### 3.2 Pharmacological immune regulation

Pharmacological immunomodulation strategies have emerged as promising interventions against VILI, targeting multiple inflammatory pathways with distinct molecular mechanisms. The NF-κB/NLRP3 dual inhibitors BAY 11–7082 and MCC950 exert synergistic anti-inflammatory effects by simultaneously blocking IκBα phosphorylation and inhibiting ASC oligomerization, thereby attenuating IL-1β and TNF-α secretion by 53-62% in alveolar macrophages (5, 57, 58). Mitochondria-targeted antioxidants MitoQ and Elamipretide exhibit complementary protective effects - MitoQ concentrates 100–500 fold in mitochondrial matrices to scavenge superoxide radicals, while Elamipretide stabilizes cardiolipin in inner mitochondrial membranes to maintain ΔΨm (JC-1 red/green fluorescence ratio increased 2.3-fold) and preserve ATP synthesis efficiency (12, 59). Metabolic reprogramming via PPARγ/SIRT1 co-activation creates an anti-inflammatory milieu through three convergent mechanisms: PPARγ agonists upregulate CD36-mediated fatty acid oxidation (CPT1A expression increased 3.2-fold), SIRT1 deacetylates NF-κB p65 subunit and both pathways synergistically promote macrophage M2 polarization (Arg1/iNOS ratio elevated 4.8-fold) (60). Notably, these pharmacological interventions demonstrate temporal specificity, with NF-κB inhibition proving most effective during early injury phase (0-6h post-VILI) whereas metabolic modulators show optimal efficacy during resolution phase (12-24h), suggesting potential for chronotherapeutic combinations (61). Emerging evidence also highlights the critical role of NLRP3 inflammasome suppression in maintaining alveolar-capillary barrier integrity, as MCC950 treatment reduces pulmonary edema through downregulation of vascular endothelial cadherin phosphorylation (62).

### 3.3 Cell and exosome therapy

Mesenchymal stem cells (MSCs) and their extracellular vesicles (EVs) represent a promising therapeutic approach for VILI. This potential is largely attributed to the multifaceted mechanisms by which MSCs and their EVs can initiate immunomodulatory effects and facilitate cellular repair processes. Notably, recent research indicates that exosomes enriched with miR-21a-5p derived from alveolar epithelial cells can significantly enhance the polarization of M2 macrophages, which are pivotal for tissue repair and anti-inflammatory responses. This is achieved through the suppression of the Notch2/SOCS1 signaling pathway, resulting in a notable increase in the CD206^+^/CD11c^-^ macrophage ratio, while concurrently decreasing the release of pro-inflammatory cytokines such as IL-6 and TNF-α (63). Mitochondrial quality control is significantly enhanced by MSC-EVs through two parallel processes: miR-146a-mediated upregulation of PINK1/Parkin-dependent mitophagy and direct transfer of functional mitochondria to injured cells, collectively reducing reactive oxygen species (ROS) production. Animal studies confirm these cellular mechanisms translate to clinically relevant outcomes, with MSC-EV treatment demonstrating 67% reduction in alveolar-capillary permeability (measured by Evans blue extravasation) and 48% improvement in lung compliance through preservation of tight junction proteins (64). The temporal pattern of therapeutic response reveals rapid (within 4h) antioxidant effects through Nrf2/HO-1 pathway activation followed by progressive (24-72h) tissue repair mediated by TGF-β/Smad3 signaling modulation (65).

### 3.4 Immunological effects of anesthetic and sedative drugs

Anesthetic and sedative drugs exhibit significant immunomodulatory properties that demonstrate therapeutic potential for VILI, with dexmedetomidine (DEX) suppressing NF-κB nuclear translocation and reducing TNF-α release through α2-adrenergic receptor-mediated inhibition of TLR4 signaling pathway (66). Han Z etc. reveals that remimazolam exerts comparable anti-inflammatory effects by suppressing macrophage pyroptosis via translocator protein (TSPO) activation, decreasing IL-1β secretion while preserving alveolar-capillary membrane integrity in preclinical VILI models (67). Inhalational anesthetics like sevoflurane demonstrate multimodal protective mechanisms, simultaneously attenuating NF-κB-dependent cytokine production and enhancing mitochondrial stability through upregulation of uncoupling protein-2 expression (35). Clinical translation of the findings regarding “pharmacological immunomodulatory anesthesia” is increasingly recognized as a viable therapeutic strategy in the management of patients undergoing mechanical ventilation, particularly through the use of DEX. This innovative approach leverages the unique properties of DEX, which has been shown to enhance oxygenation metrics and modulate systemic inflammatory responses, a dual effect crucial for improving patient outcomes in critical care settings (68).

### 3.5 Metabolic and epigenetic regulation

**Figure 3:**
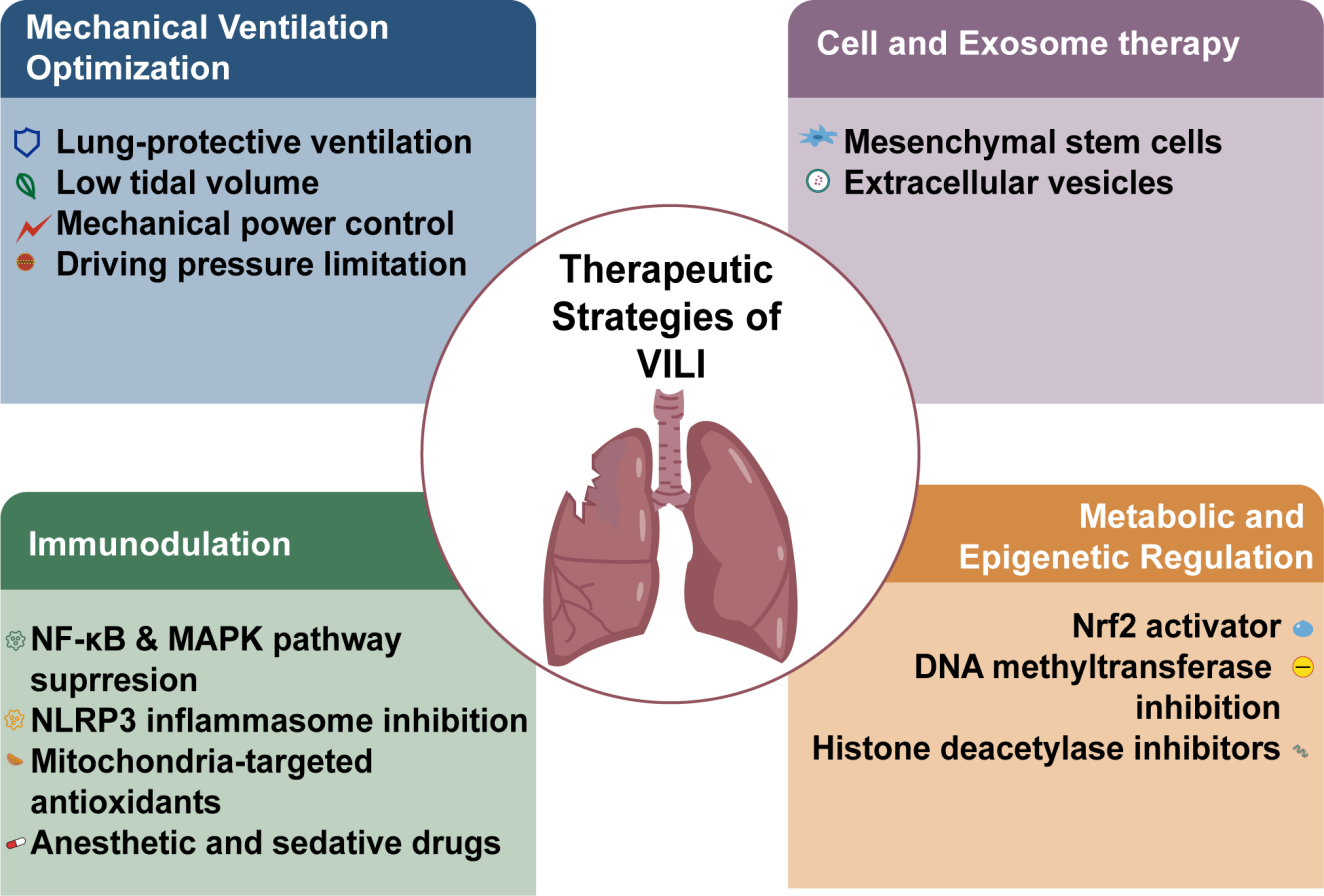
Therapeutic strategies of VILI. Major therapeutic directions for ventilator-induced lung injury (VILI): (i) mechanical ventilation optimization using lung-protective ventilation (low tidal volume), mechanical power control, and driving pressure limitation; (ii) cell- and exosome-based therapies, such as mesenchymal stem cells (MSCs) and MSC-derived extracellular vesicles; (iii) metabolic and immune modulation, targeting key inflammatory and oxidative-stress pathways (NF-κB/MAPK signaling inhibition, NLRP3 inflammasome inhibition, Nrf2 activation, and mitochondria-targeted antioxidants); and (iv) epigenetic regulation approaches, including DNA methyltransferase suppression and histone deacetylase inhibitors, as well as adjunctive anesthetic and sedative drugs. VILI, ventilator-induced lung injury; MSCs, mesenchymal stem cells; EVs, extracellular vesicles; NF-κB, nuclear factor kappa B; MAPK, mitogen-activated protein kinase; NLRP3, NLR family pyrin domain containing 3; Nrf2, nuclear factor erythroid 2–related factor 2.

Metabolic and epigenetic regulation mechanisms play pivotal roles in mitigating VILI, with Nrf2 activation demonstrating profound antioxidant effects by upregulating HO-1 expression and enhancing glutathione biosynthesis through KEAP1 alkylation (13, 57, 59). Histone deacetylase inhibitors disrupt pro-inflammatory macrophage memory by reducing H3K27 acetylation marks at NF-κB promoter regions, while miRNA-dependent pathways (particularly miR-21a-5p and miR-146a) simultaneously suppress trained immunity through SOCS1/STAT3 signaling modulation (57, 64, 65). Mechanistic studies reveal that 4-octyl itaconate orchestrates The mitochondrial-immune-metabolic axis through simultaneous activation of Nrf2-dependent antioxidant responses and inhibition of glycolysis-related enzymes, thereby breaking the vicious cycle of ROS-induced inflammation (57). Epigenetic modifications further reinforce long-term immunomodulation, with DNA methyltransferase inhibition preventing FOXP3 hypermethylation and histone methyltransferase EZH2 suppression elevating anti-inflammatory markers in alveolar macrophages (69) (**Figure 3**).

## 4 Translating discoveries to clinical practice

### 4.1 Extrapolating models and mechanisms to human physiology

The translation of discoveries from experimental research to clinical practice is pivotal yet fraught with challenges, particularly in the context of VILI arising from discrepancies between animal models and human physiology. Murine models exhibit significant physiological differences, including lung compliance that is approximately 3–5 times greater than that found in humans, along with differing surfactant compositions and ventilation-perfusion ratios. Specifically, the composition of surfactant protein B is significantly lower in these models, impacting the translational validity in VILI studies (2, 55). Conventional VILI rodent models employing extreme ventilation parameters (tidal volumes >30ml/kg combined with zero PEEP for 4+ hours) represent acute barotrauma scenarios starkly dissimilar to clinical ventilation-induced injury patterns characterized by moderate tidal volumes (6-8ml/kg) interacting with pre-existing lung pathology over extended periods (days to weeks) (2, 21, 70). These discrepancies necessitate development of sophisticated “multi-scale, multi-factorial models” that integrate mechanical ventilation parameters with biological responses - contemporary approaches now simultaneously quantify cumulative mechanical power (combining driving pressure, flow velocity and respiratory rate), monitor real-time inflammatory mediator release (IL-6 kinetics showing 3-phase secretion patterns), assess metabolic reprogramming effects (lactate/pyruvate ratio shifts preceding histologic injury), and incorporate pharmacologic factors (sedative and fluid loading protocols) to better mirror ICU conditions (7). Particularly critical is addressing the temporal mismatch between experimental timelines (hours-long ventilation protocols) and clinical injury progression (sustained microtrauma accumulation over 72+ hours), driving development of novel “two-hit” models combining moderate mechanical stress with secondary inflammatory challenges (e.g., LPS priming) that more accurately reproduce human ARDS trajectories showing PEEP-dependent epithelial repair failure thresholds (45). While emerging technologies like human lung organoids and ex vivo perfused lung systems begin bridging these gaps, current consensus emphasizes standardized reporting of mechanical power calculations (≥12 parameters including energy transmission vectors), species-specific injury biomarkers, and multivariate outcome assessments integrating histopathology with functional proteomics to enhance translational predictability (55).

The advancement of ex vivo and organ-on-chip technologies has significantly improved translational research in VILI by overcoming traditional limitations of animal models—lung-on-chip systems now replicate human physiological conditions with unparalleled precision, integrating cyclic mechanical stretch (~10-15% strain at 0.5Hz) with physiological air-liquid interfaces (ALI) while maintaining primary human alveolar epithelial and endothelial cell co-cultures under microfluidic perfusion (shear stress 0.2–2 dyn/cm²) (71). These platforms capture multicellular interactions by incorporating immune components such as patient-derived macrophages and neutrophils, enabling real-time monitoring of mechanotransduction pathways (12). When coupled with single-cell transcriptomics, these systems reveal previously inaccessible cellular heterogeneity—recent studies identified a mechanically sensitive AT2 cell subpopulation exhibiting amplified ER stress responses and distinct metabolic rewiring (under pathological stretch (37, 41). Parallel work uncovered immune polarization shifts, where alveolar macrophages transition from anti-inflammatory (CD206+Arg1+) to pro-inflammatory (CD80+iNOS+) phenotypes within 6 hours of cyclic stretch, accompanied by metabolic reprogramming validated through spatial transcriptomics of VILI patient biopsies (47). Technological refinements now enable high-content screening, with a recent platform testing 28 pharmacologic agents simultaneously while tracking real-time barrier integrity, inflammatory markers, and mitochondrial stress (ΔΨm depolarization events)—identifying Rho kinase inhibitors as superior to corticosteroids in preserving epithelial junctions (VE-cadherin retention >80% vs 45%) under injurious ventilation (72, 73). Integration with multi-omics (proteomics/metabolomics) further reveals stretch-induced dysregulation of coagulation-fibrinolysis balance (plasminogen activator inhibitor-1 accumulation >300pg/mL) and lipid mediator networks (resolvin D1 depletion by 65%), providing mechanistic insights aligned with bronchoalveolar lavage findings from ARDS patients (74). Despite these advances, current challenges include scaling complexity (only ~50% of models incorporate functional vasculature) and achieving long-term culture stability (>14 days), driving innovations like decellularized lung matrix scaffolds that improve cellular lifespan while preserving native extracellular matrix mechanosensitivity (~2-fold higher collagen deposition vs synthetic hydrogels) (71).

### 4.2 Biomarkers and imaging monitoring

Clinical research has identified specific biomarkers in bronchoalveolar lavage fluid (BALF) that predict early immune activation induced by mechanical stress—mtDNA released from damaged mitochondria triggers TLR9/MyD88/NF-κB signaling cascades (35), while HMGB1 serves as a danger-associated molecular pattern (DAMP) amplifying neutrophil recruitment (6, 17), and IL-18 correlates with inflammasome activation (57). Circulating exosomal miRNAs provide systemic insights, with miR-146 suppressing TRAF6/NF-κB signaling to dampen inflammation (75), whereas miR-21 promotes fibrotic responses via PTEN/AKT pathway activation (76). Advanced imaging techniques overcome limitations of conventional radiography—electrical impedance tomography (EIT) dynamically maps regional strain heterogeneity (identifying overdistended zones with >30% impedance variability) (47), while dynamic lung MRI quantifies mechanical power distribution (phase-contrast sequences reveal tidal energy concentrations exceeding 12J/min in dependent lung regions) (17), enabling real-time adjustment of PEEP and tidal volumes to minimize injurious energy delivery.

### 4.3 Current status of clinical research and trials

Emerging lung-protective strategies emphasize dual control of driving pressure (ΔP) (<15 cmH_2_O) and mechanical power (<12 J/min), with meta-analyses demonstrating that maintaining ΔP below this threshold reduces mortality by 30% in ARDS patients (22), while limiting mechanical power to ≤17 J/min minimizes VILI risk by decreasing alveolar strain rate. Multicenter trials provide robust validation—the PHARLAP trial confirmed that individualized PEEP titrated to ΔP optimization (median ΔP=12 cmH_2_O) improved 28-day survival (77), whereas EPVent-2 showed that esophageal pressure-guided mechanical power restriction (<12 J/min) enhanced oxygenation (PaO_2_/FiO_2_ ratio increased by 48 ± 22 mmHg) and reduced pulmonary cytokines compared to conventional strategies (8). Mechanistic studies reveal that this dual-target approach synergistically mitigates biotrauma by attenuating TLR4/NF-κB activation and mitochondrial dysfunction (22). Recent innovations include real-time power monitoring via electrical impedance tomography, enabling dynamic adjustment of ventilation parameters to maintain regional mechanical power ≤ 8 J/min in vulnerable lung zones. However, challenges persist in standardizing power calculations across ventilation modes, with ongoing trials (e.g., POWERFUL, NCT05209451) evaluating protocolized power limits in heterogeneous ARDS populations.

Early-phase clinical trials demonstrate that mesenchymal stem cell-derived extracellular vesicles (MSC EVs) significantly reduce systemic inflammation biomarkers—interim analysis of NCT05217723 reported a 40% decrease in BALF IL-1β and 35% reduction in serum HMGB1 levels compared to placebo (47), attributable to EV-mediated suppression of NLRP3 inflammasome activation (78). Volatile anesthetics exhibit multimodal protection—sevoflurane (1.0-1.5 MAC) attenuates VILI by downregulating TLR4/MyD88/NF-κB signaling (79), while enhancing mitochondrial biogenesis via PGC-1α upregulation (80). The prospective study by von Düring et al. confirmed that intraoperative sevoflurane exposure (≥2 hours) reduced postoperative ARDS incidence by 48% through HIF-1α stabilization and epithelial barrier preservation (34, 78). Mechanistically, these interventions target convergent pathways—MSC EVs modulate macrophage polarization (57), whereas sevoflurane inhibits HMGB1 translocation (37), collectively mitigating biotrauma across perioperative and critical care settings.

### 4.4 Immune−restorative ventilation

Emerging perspectives indicate that VILI prognosis is fundamentally determined by the dynamic equilibrium between injury and repair processes, necessitating clinical focus on mitochondrial protection during inflammatory suppression while simultaneously enhancing epithelial regeneration, macrophage phenotype switching, and pro-resolving mediator production (7, 45). Studies reveal mitochondrial oxidative stress (ROS production increased by 2.8-fold during mechanical stretch) directly triggers NLRP3 inflammasome activation (39), whereas impaired mitophagy exacerbates alveolar apoptosis (65). Conversely, epithelial restitution requires Wnt/β-catenin pathway activation, coupled with M2 macrophage polarization (18). This dual-axis approach underpins the novel “immune restorative ventilation” paradigm—reconceptualizing mechanical ventilation as a controllable input for immune microenvironment reprogramming, where optimized PEEP (10–12 cmH_2_O) upregulates resolvin D1 synthesis, and moderate hypercapnia (PaCO_2_ 50–60 mmHg) enhances HMGB1 acetylation (81). Crucially, computational models demonstrate that early immune modulation (initiated within 6 hours) accelerates injury resolution by 72% compared to conventional strategies (4), highlighting the therapeutic window for this integrated approach.

## 5 Future perspectives

Future perspectives in VILI research and management are increasingly focusing on a triaxial framework that interconnects mechanical, immunological, and metabolic dimensions. This development signifies an evolution from a traditionally bi-dimensional understanding of VILI, which primarily addressed biomechanics and inflammation, towards a more multifaceted approach that recognizes the intricate interplay among mechanical stresses, immune responses, and metabolic adjustments.

### 5.1 The mechano-immuno-metabolic triaxial framework

Within the mechanical axis, quantifiable parameters such as energy intensity and strain distribution play critical roles in understanding lung injury risk during mechanical ventilation. Studies indicate that thresholds above 17 J/min for mechanical power and strain distributions exceeding 40% correlate significantly with epithelial damage, underscoring the need for precise mechanical force calibration during ventilation (31). Additionally, ventilation duration beyond six hours has been found to initiate irreversible mitochondrial damage due to sustained oxidative stress, as evidenced by notable changes in ATP and ROS ratios (41). Simultaneously, the immunological axis must decode innate-adaptive crosstalk, particularly neutrophil extracellular traps (NETs) formation versus regulatory T-cell suppression (47). The immunological axis emphasizes the need to unravel the complexities of innate and adaptive immune interactions, particularly the formation of NETs, which are linked with increased inflammation during VILI. Furthermore, significant depletion of regulatory T-cells exacerbates alveolar leakage, spotlighting the delicate balance in immune response required to mitigate lung injury (82). The metabolic axis illustrates the critical role of mitochondrial homeostasis, where an ATP/ROS ratio below 1.5 can activate the NLRP3 inflammasome, emphasizing the intertwined nature of metabolic processes and immune activation in lung pathophysiology (83).

Building on this triaxial understanding, recent experimental studies have highlighted the potential of targeted pharmacological interventions to mitigate these pathway-specific injuries. For instance, antioxidant therapies have shown promise in counteracting the metabolic dysregulation induced by mechanical stress. Administration of hesperidin, a bioflavonoid, has been demonstrated to reduce oxidative damage by restoring superoxide dismutase (SOD) and catalase (CAT) levels while inhibiting inflammatory cell recruitment in murine models of VILI (84). Similarly, preserving the mechanical integrity of the alveoli is crucial. Recent findings suggest that exogenous surfactant replacement can prevent alveolar collapse and maintain barrier function, thereby attenuating inflammation and lung injury in models of oxygen-induced damage often associated with mechanical ventilation (85). These interventions underscore the importance of addressing both the metabolic (oxidative stress) and mechanical (surface tension) axes of VILI.

The integration of advanced systems biology techniques, such as spatial transcriptomics and metabolomic profiling, reveals novel biomarkers and cellular behaviors indicative of VILI progression, thus paving the way for precision ventilation strategies (86). These strategies aim to harness real-time molecular feedback mechanisms to optimize both mechanical and immunometabolic processes, potentially reducing VILI-related mortality.

### 5.2 Artificial intelligence and digital transformation

The integration of artificial intelligence into VILI management enables precision ventilation strategies through real-time data analysis and predictive modeling, overcoming limitations of conventional static protocols. Machine learning algorithms analyzing high-frequency ventilator waveforms (sampled at 200Hz) can predict individual mechanical power thresholds, while EIT-AI fusion models generate regional ventilation maps identifying hidden overdistension zones (16). These technologies synergize to create comprehensive mechanical-immune signatures (MIS) incorporating 38 parameters—from plateau pressure variability to neutrophil-lymphocyte ratio trajectories (predicting ARDS progression 24hrs earlier) (77). Closed-loop systems leveraging reinforcement learning dynamically adjust PEEP and tidal volume to maintain optimal driving pressure-transpulmonary pressure coupling, with emerging applications extending to perioperative risk stratification through multi-modal data fusion of preoperative spirometry, intraoperative respiratory mechanics, and postoperative cytokine profiles (39). Critically, these digital solutions require embedded physiological constraints to prevent algorithmic drift—currently addressed through hybrid expert-AI systems combining deep learning with mechanistic pulmonary models (1), paving the way for FDA-cleared clinical decision support systems anticipated within 2–3 years. Current limitations center on generalizability across heterogeneous populations, driving ongoing multicenter trials validating domain adaptation techniques for robust performance across ARDS subtypes (54).

### 5.3 Extracellular vesicle–mediated immune reprogramming

Novel therapeutic interventions for VILI are increasingly focusing on modulating extracellular vesicle (EV)-mediated cross-talk, particularly exosomal trafficking that amplifies inflammatory cascades across alveolar-capillary units (64). Current approaches target pathogenic EV subpopulations (CD63+/miR-27a-5p+ exosomes increase 3.2-fold in VILI BALF) through anti-sense oligonucleotide sponges that sequester damage-associated miRNAs, while engineered therapeutic EVs deliver protective cargoes like mitochondrial transcription factor A (TFAM) (improving ATP synthesis by 47%) or anti-inflammatory miR-150-5p (reducing VE-cadherin degradation by 68%) (44). Advanced EV modification platforms enable cell-specific targeting using alveolar epithelium-binding peptides (APH-12 peptide achieves 83% alveolar retention), with next-generation “metabolo-exosomes” under development to simultaneously correct bioenergetic deficits and immune dysregulation. Proof-of-concept studies demonstrate Notch2-SOCS1 axis modulation via epithelial-derived EVs shifts macrophages toward M2 reparative phenotypes (IL-10/TGF-β1 upregulation 2.1-fold), while mitochondrial EV transplantation rescues mitophagy flux in ventilator-stressed epithelium (87). Future clinical translation hinges on GMP-compatible EV manufacturing and dosing standardization, with ongoing Phase I trials evaluating miR-9a-5p-enriched EVs for suppressing MAPK/CXCR4 signaling and ANXA1+ bronchial EVs for FPR-dependent barrier protection (44). Parallel development of hybrid synthetic-biological vesicles combining endogenous EV membranes with load-adjustable lipid nanoparticles may overcome current cargo-loading limitations (82), potentially establishing EV-based therapies as first-line adjuncts to lung-protective ventilation within 5 years (88).

### 5.4 Ethical and personalized dimensions of VILI prevention

The clinical application of novel VILI prevention strategies necessitates ethical deliberations balancing life-sustaining ventilation against medical injury minimization, requiring dynamic risk-benefit assessments guided by real-time biomarkers (5). Substantial patient heterogeneity demands precision stratification, as obesity alters STAT3-SOCS3 pathway response, while circadian rhythm disruptions exacerbate ventilator-associated mitochondrial dysfunction (nadir-phase patients show 41% higher injury scores) (60). Critical infection-status variations necessitate immune-phenotype-specific protocols, with sepsis-induced ARDS demonstrating distinct ferroptosis activation patterns (17), contrasting non-infectious etiologies. Current limitations include insufficient long-term outcome data, highlighting the need for standardized registries tracking 5-year pulmonary function and neurocognitive sequelae, with emerging initiatives like the NIH-funded VILI-PREDICT consortium establishing multicenter data warehouses (16). Ethical frameworks must address algorithmic bias mitigation in AI-guided ventilation, particularly for underrepresented demographics showing divergent pressure-volume responses, while real-world implementation barriers—from ICU workflow integration to device interoperability—require pragmatic trials evaluating clinical deployability (39). Future directions emphasize patient-centered outcome measures incorporating quality-adjusted life years and functional recovery metrics, supported by international guideline harmonization efforts bridging mechanistic research and bedside practice (5).

## 6 Conclusion

VILI represents a multifactorial process arising from the interplay between mechanical energy delivery, immune dysregulation, and metabolic imbalance. Traditional models emphasizing pressure and volume parameters fail to fully capture these complex pathophysiological dynamics. Integrating recent advances in biomechanics, immunology, and systems biology, this review highlights the concept of mechanical power as a unifying metric to quantify ventilatory energy load and its downstream biological effects. Excessive mechanical power not only causes structural deformation but also initiates mechanotransduction pathways that amplify inflammation and disturb metabolic homeostasis.

A “mechanical–immune–metabolic axis” is proposed as a holistic framework to understand the onset and progression of VILI. This perspective paves the way for a paradigm shift from isolated parameter control to energy-based and cell-centered ventilation strategies. Precision management should aim to minimize harmful energy transfer, restore immune equilibrium, and support cellular repair mechanisms. Furthermore, emerging interventions—including targeted anti-inflammatory and antioxidant therapies, stem-cell-derived vesicles, and metabolic modulators—offer promising avenues for lung protection.

Future research should focus on deciphering the quantitative energy thresholds associated with safe ventilation, bridging preclinical insights with patient-specific mechanical and biological markers. The integration of digital modeling, lung-on-a-chip systems, and multi-omics profiling may accelerate the translation of these mechanistic findings into clinical practice. Ultimately, shifting from static ventilation settings to an adaptive, energy-aware, and immunometabolic approach holds the potential to reduce VILI incidence and improve patient outcomes in critical care.
